# Maternal complications in twin pregnancies in Finland during 1987–2014: a retrospective study

**DOI:** 10.1186/s12884-019-2498-x

**Published:** 2019-09-18

**Authors:** Annu-Riikka S. Rissanen, Riina M. Jernman, Mika Gissler, Irmeli Nupponen, Mika E. Nuutila

**Affiliations:** 1 Obstetrics and Gynecology, University of Helsinki and Welfare District of Päijät-Häme, Keskussairaalankatu 7, 15850 Lahti, Finland; 2 Obstetrics and Gynecology, University of Helsinki and Helsinki University Hospital, Haartmaninkatu 2, P.O. BOX 140, 00029 HUS Helsinki, Finland; 3 Finnish Institute for Health and Welfare, P.O. BOX 30, 00271 Helsinki, Finland; 4 Karolinska Institute; Department of Neurobiology, Care Sciences and Society, Stockholm, Sweden; 5 Children’s Hospital, University of Helsinki and Helsinki University Hospital, Stenbäckinkatu 11, P.O. BOX 281, 00029 HUS Helsinki, Finland

**Keywords:** Twins, Multiple pregnancy, Register data

## Abstract

**Background:**

To investigate the trends and changes in the incidence and overall outcome of twin pregnancies in Finland, a retrospective study was conducted with emphasis on maternal complications, covering a 28-year study period.

**Methods:**

All 23,498 twin pregnancies with 46,363 live born and 633 stillborn children in Finland during 1987–2014 were included in the study. Data were collected from the national Medical Birth Register and the Care Register on Hospital Care (Finnish Institute for Health and Welfare, Finland) regarding the parturients’ characteristics and incidences of several pregnancy and childbirth complications. The incidences of twin pregnancies and maternal complications during pregnancy and childbirth are the main outcome measures of the study. The results are expressed in percentages, means, medians, ranges and standard deviations (SD), when appropriate.

**Results:**

Twins comprised 1.4% of all births in Finland in 1987–2014. Parturients’ mean age has remained stable, but the share of over 35 year-old parturients is increasing. The incidences of pre-eclampsia, intrahepatic cholestasis of pregnancy, gestational diabetes and postpartum haemorrhage have risen during the study period. Almost half (44.9%) of twins were born preterm, almost half via Caesarean section (47.1%), and 27.7% of twin labours were induced.

**Conclusions:**

Several pregnancy complications increased during the study period. Advanced maternal age among twin parturients has risen, enhancing the risks for developing complications in a pregnancy already of a high-risk category, and predisposing to preterm delivery. National and international guidelines are necessary to improve the overall outcome of twin pregnancies.

## Background

Twin pregnancy involves several risks for the mother and the offspring. Preterm delivery and pre-eclampsia are increased, but data are conflicting whether the risk of gestational diabetes (GDM) is also higher [1–4]. Problems in childbirth are more common than with singletons, and roughly half of twins are born via Caesarean section (CS) [5]. Parturients’ increasing age and body mass index add to the risk for developing complications and delivering by CS [6, 7]. Increased blood loss and thromboembolism may also arise more often [8, 9].

Assisted reproductive technology (ART), such as multiple embryo transfer or ovarian stimulation, increases the number of multiple gestations. These are iatrogenically produced high-risk pregnancies that should be avoided [10, 11]. Treatment policies and outcomes may differ markedly by country and clinic ([12].

The main concern with twins is prematurity and its consequences. Perinatal mortality is generally higher among twins, although in preterm newborns it has been reported to be lower than in singletons in the corresponding gestational age [13, 14].

The population in Finland is rather homogenous, considering both ethnicity and maternal care, and all twin pregnancies and deliveries are managed in public hospitals. No national guidelines, however, exist for twin pregnancies and hospital districts follow their own protocols. Our purpose was to establish the trends and changes in the overall outcome of twin pregnancies in Finland during a 28-year study period, focusing here on maternal complications. Data on twin pregnancies in Finland have previously not been reported to this extent.

## Methods

This retrospective study consists of all 23,498 twin pregnancies with 46,363 live births and 633 stillbirths in Finland during 1987–2014. The data were collected from the national Medical Birth Register and the Care Register on Hospital Care (Finnish Institute for Health and Welfare, Finland). The Medical Birth Register contains, from 1987 onwards, data on all births in Finland regarding information on parturients, deliveries and newborns until the age of seven days. The Care Register includes data on hospital treatment received including diagnoses, procedures and interventions. From these registers we separated diagnoses (International Statistical Classification of Diseases and Related Health Problems, ICD-9 in 1987–1995, ICD-10 since 1996) and operations (Nordic Medico-Statistical Committee Classification on Surgical Procedures) on twin mothers. In the Medical Birth Register, data on ART is available since 1990, induction of labour and parturients who quit smoking since 1991, parturients’ body mass index, obesity and pregnancy-induced hypertension (PIH) since 2004. Several other pregnancy complications and existing diagnoses (such as hypertensive and diabetic complications; intrahepatic cholestasis of pregnancy, intrahepatic cholestasis of pregnancy; placentation problems; postpartum haemorrhage, PPH; perineal tears; and thromboembolic complications) is available since 1996 after combining the two registers.

The following data were analysed: the incidence of twin pregnancies (spontaneous /ART), parturients’ age, parity, body mass index, smoking, PIH, pre-eclampsia, gestational diabetes (GDM), intrahepatic cholestasis of pregnancy, preterm deliveries, the onset of labour (spontaneous/induced), mode of delivery, perineal/vaginal tears, massive PPH, thromboembolic events, maternal deaths, time of delivery and perinatal mortality. Perinatal mortality was defined as death in the perinatal period (from 22 weeks of gestation up to 6 days postpartum) per 1000 children born (dead or alive). The number of all CS is reported by CS performed for twin B, despite the delivery method for twin A. Urgent CS includes all non-elective CSs with a decision-to-delivery interval from 10 min (emergency CS) to 30 min or more.

The following data were collected by ICD-10 and corresponding ICD-9 codes: PIH (O13), pre-eclampsia (O14.0, O14.1, O14.9), GDM (O24.4), intrahepatic cholestasis of pregnancy (O26.6), preterm contractions (O47.0), perineal tears (O70.0, O70.1, O70.2, O70.3, O70.9), PPH (O72.0, O72.1, O72.2, O72.3) thromboembolic events (O22.3, O22.5, O22.8, O22.9, I26), and maternal deaths (O95, O96, O97). The data were separated for twin A and B when possible and compared to separately retrieved singleton data, when necessary. We were unable to separate chorionicity, as it was not recorded in the registers during the study period. In-depth analysis of the outcome of the offspring including further details on perinatal mortality and chorionicity will be covered in our future reports.

With the data provided, we present the trends and changes in the maternal complications of twin pregnancies in Finland.

### Statistical analyses

The data were analysed using SPSS (IBM SPSS Statistics for Windows, Version 24.0, Armonk, NY, IBM Corporation). Assessment of the normal distribution of variables was done with the Shapiro-Wilk test and a *p*-value <0.05 with a 95% confidence interval was considered statistically significant. To compare medians of the variables, related samples Wilcoxon signed rank test was used. One sample t-test was used to compare means of variables. Microsoft Excel 2010 and SPSS 24.0 were used to create figures, graphs and trend lines. The results are expressed in percentages, and the means, medians, ranges and standard deviations (SD) are reported when appropriate.

## Results

**Fig. 1:**
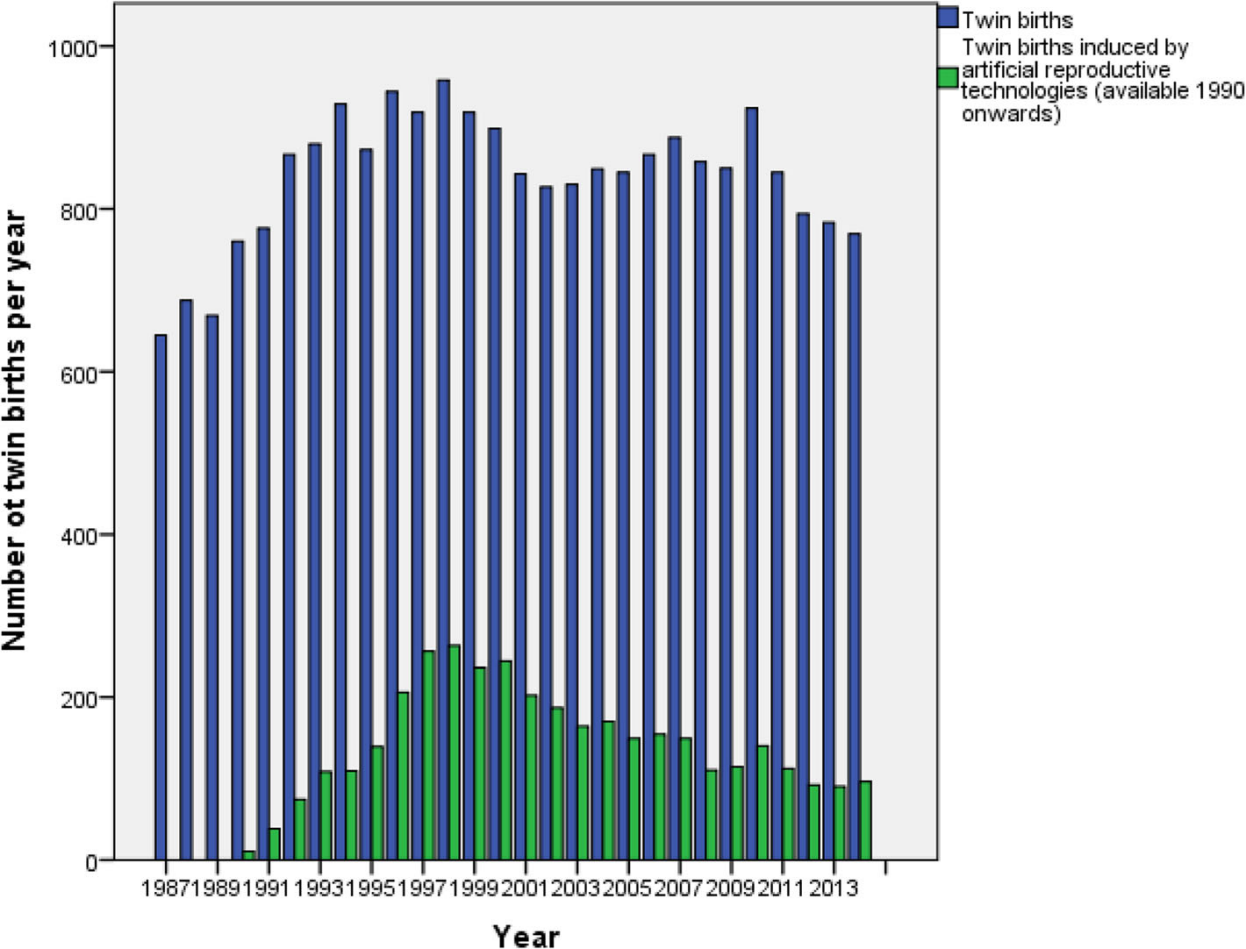
The number of spontaneous and artificial reproductive technology-induced twin births in Finland during 1987–2014. Data on ART-induced twin pregnancies is available from 1990 onwards, reported per year

During the years 1987–2014 there were 23,498 twin pregnancies in Finland (mean 839 per year; range 631–950) (Fig. 1). Twin pregnancies accounted for 1.4% of all births in Finland, increasing from the lowest 1.1% in the late 1980s to the highest 1.7% in 1998. The increase in the incidence of twins was simultaneous with the enhanced use of ART, as the number of ART-induced twin pregnancies peaked in the late-1990s, being highest in 1997 (27.9% of twin pregnancies). Afterwards, it has steadily decreased and stabilized 2008 onwards to approximately 12.9% of all twin births.

The mean age of twin parturients was 30.9 years (±5.1 SD, range 29.7–31.6) and of twin nulliparas 29.7 years (±5.1 SD, range 27.4–30.7). In both groups the mean age has steadily increased during the study period, although slightly slower from 1995 onwards. In parturients over 35 years of age, defined as advanced maternal age, the rise has been more marked. The share of mothers with advanced maternal age began to increase in 1992 from 16 to 29% in 2003, after which it decreased to 22–24% and from 2012 onwards, has accounted for 26% of twin parturients. This is significantly higher (*p* < 0.001) compared to singletons (range 13.2–20.3% from 1987 to 2014), Fig. 2. The share of under 20 year-olds old has remained stable throughout the study period (1.5%).

**Fig. 2:**
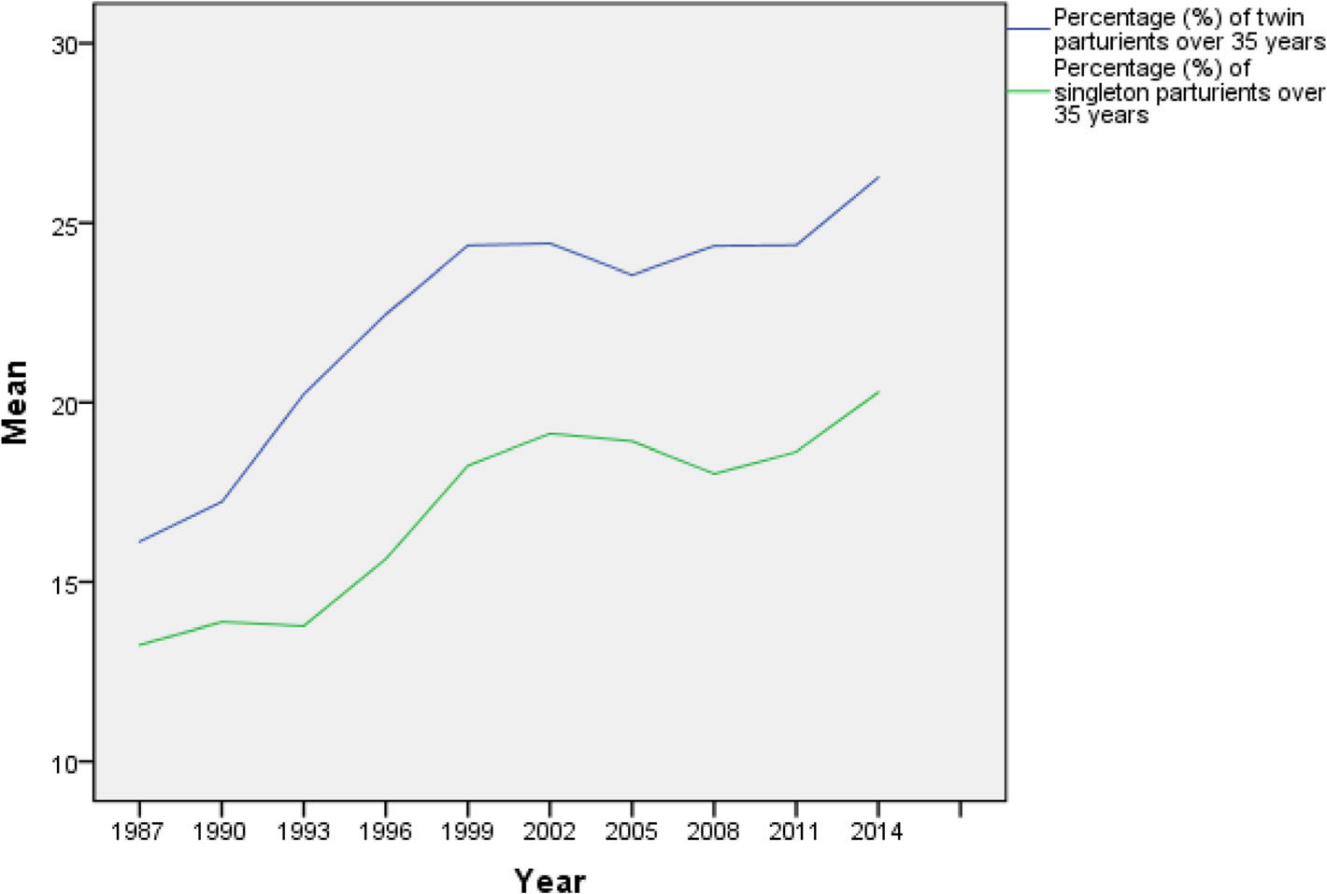
The shares of twin and singleton parturients over 35 years during 1987–2014. The data is presented at three-year intervals

Most twin parturients were nulliparas; GXP0 43.0% and G1P0 31.7%. These prevalences increased in the early 1990s and started to decline slightly in 2005. Almost one fifth (19.1%) had had at least three preceding pregnancies (G4 or more); 8.8% had had three or more deliveries (P3 or more), and 21.9% had had a previous miscarriage. The latter number has increased in the last eight years of the study period. Among singletons, 40.8% of parturients were nulliparas.

Mean body mass index was 24.6 kg/m^2^ (±4.9 SD, range 24.1–25.1) and has been relatively stable and similar compared to singletons (±4.8 SD range 24.1–24.6). Obesity rose from the first reported 0.4% in 2004 to 3.1% in 2014, but the ICD-10 definition may vary. Among twin parturients, 13.8% continued smoking during pregnancy. The proportion who reported quitting smoking increased from 1.0% in 1991 (the first year reported) to 5.9% in 2014, and was highest in 2012 (6.6%).

**Fig. 3:**
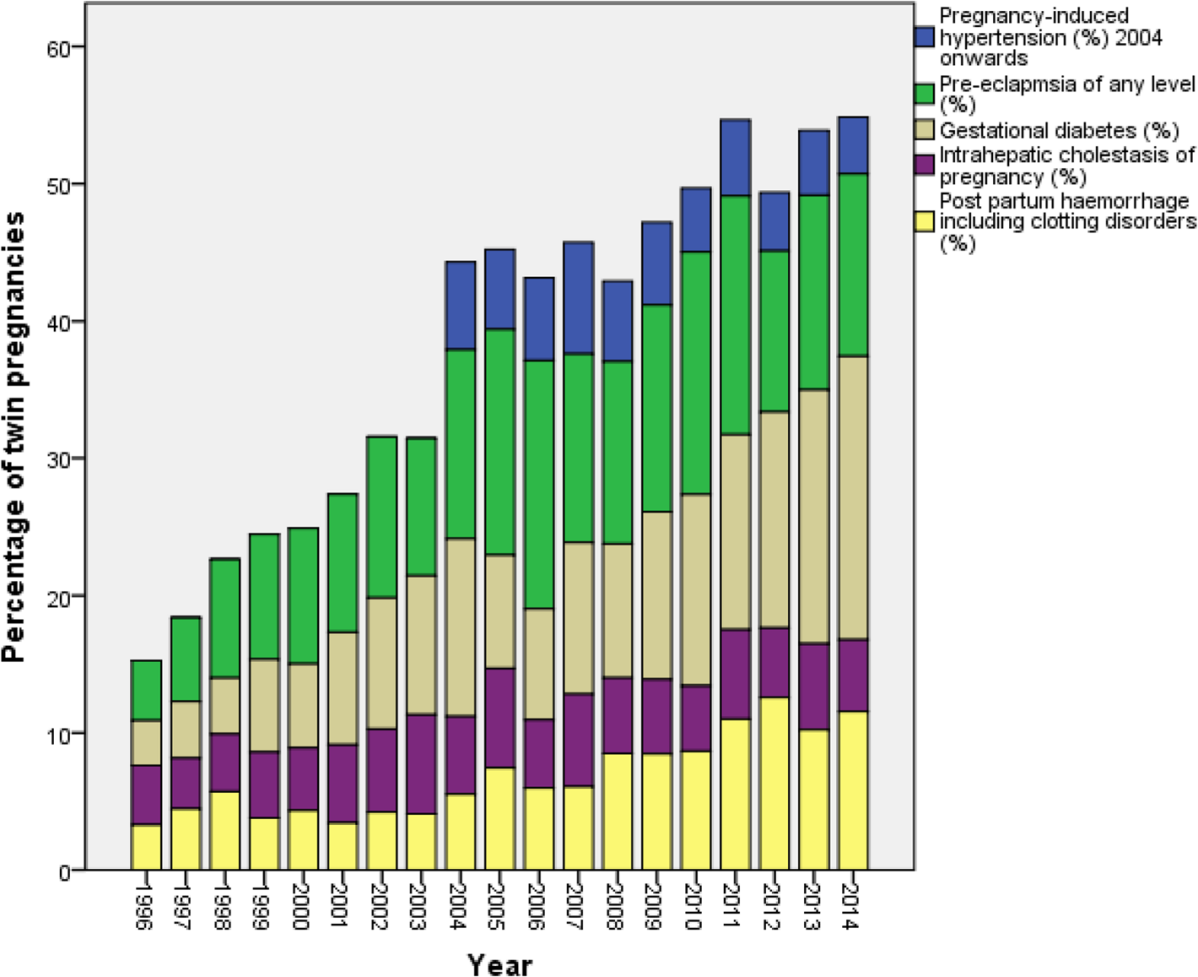
The changes in the incidences of pregnancy complications in twin pregnancies in Finland during 1996–2014. Information on pregnancy induced hypertension is available from 2004, data on other complications from 1996 onwards. The bars represent percentage of twin pregnancies with respective pregnancy complications

The incidence of pre-eclampsia (range 4.3–18.1%) increased significantly (*p* = 0.017) from 1996 to 2006, after which it has been 13.3–17.6% (Fig. 3). Pregnancy-induced hypertension ranged from 4.2 to 8.1% during the reported years (2004–2014). The trend has, however, been slowly decreasing. Among singletons, pre-eclampsia of any level was reported to be markedly lower, only 0.8%, rising to 3.8% if PIH is included.

Gestational diabetes increased from 3.3 to 20.7% (*p* = 0.001, average 10.4%) among twins. In all parturients including singletons this increase was 6.3–11.5% (average 3.0%). Intrahepatic cholestasis of pregnancy ranged between 3.7–7.2% (mean 5.5%), which is higher than reported among singletons (0.4%).

### Time and mode of delivery and complications

During the study period, twin deliveries at 40–42 weeks decreased from 4.0 to 0.1% and deliveries at 37–39 weeks from 55.5 to 50.6%. Deliveries at 34–36 weeks increased from 25.7 to 33.3%. Deliveries before 34 weeks showed no significant change. Delivery was preterm (< 37 weeks) in 44.9% (range: 40.5–49.5%) in twins compared to 4.6% (range: 4.4–4.9%) among singletons.

In total, 27.7% (range 20.4–38.0%) of twin deliveries were induced, showing marked increase during the study period (*p* < 0.0001). Prostaglandin was used in approximately 5.7% of twin inductions with some fluctuation (lowest 2.6% in 1987, highest 9.6% in 2008). Other methods could not be analysed due to varying indications for use or missing information.

Vacuum extraction was used in 5.9% of deliveries for twin A and 6.0% for twin B, both numbers increasing and comparable to singletons (6.3%). The use of forceps remained rare and stable: 0.2% for both twins and 0.1% for singletons. Whether the delivery was vaginal instrumental (vacuum/forceps) for both twins could not be analysed. Breech delivery was reported 2.1% for twin A and 8.9% for twin B including breech extraction which is higher than among singletons (0.6%).

Twin A was born via CS in 45.3% and twin B in 47.1% A little less than half of CSs were elective (mean 21.4% until 2001, after which a decrease from 23.6 to 17.8% was seen (*p* = 0.025). Opposite to the share of elective CS, the proportion of all CS has risen during the reported years 1991–2014 (*p* = 0.003). The national average for CS is 15.3% among singletons.

Urgent CS was performed in 24.6% of cases to deliver both and in 26.4% to deliver twin B, which is higher than among singletons (8.9%). Combined vaginal-CS delivery (twin A born vaginally and twin B by urgent CS) took place on average in 1.8% of twin births per year. For further details, see Table 1. At least 29.6% were spontaneous vaginal (cephalic presentation) deliveries for both twins. When twin B was breech, deliveries were vaginal without instrumental assistance in 38.5%.

**Table 1:** Mode of delivery in twin births during 1987–2014 in Finland. The number of elective CS^a^ is available from 1991, breech deliveries and urgent CS from 1996. The figures are expressed at three-year intervals from 1991 to 2012; also the first year 1987 and last year 2014 included

Year	Elective CS %	All CS B % ^b^	Urg CS A % ^c^	Urg CS B %	Vacuum extraction A %	Vacuum Extraction B %	Forceps A %	Forceps B %	Breech A %	Breech B %	Vag. + CS combined % ^d^
1987		41.6			3.7	3.3	0.3	0.3			2.3
1991	21.3	40.3	16.8	19.1	4.5	5.2	0.5	0.5			2.3
1994	23.7	47.3	21.2	23.6	4.2	4.1	0.2	0.2			2.4
1997	23.2	48.6	24.2	25.5	5.2	4.5	0.0	0.1	2.2	8.6	1.3
2000	21.1	45.3	24.2	24.1	3.8	3.7	0.1	0.0	2.9	8.5	0.0
2003	22.0	45.8	24.0	23.7	5.1	5.5	0.1	0.1	1.6	8.2	0.0
2006	19.1	49.5	27.9	30.3	8.3	8.2	0.0	0.0	1.2	9.7	2.4
2009	20.9	49.9	27.1	28.9	9.9	9.5	0.1	0.0	4.2	9.1	1.9
2012	17.8	48.2	27.1	30.5	9.6	8.9	0.0	0.1	1.9	9.9	3.4
2014	17.8	47.2	26.9	29.4	9.9	9.1	0.1	0.1	3.0	10.9	2.5

^a^Cesarean section

^b^ the percentage of all CS in twin deliveries

^c^ the percentage of urgent (urg) CS in twin deliveries (A and B separately)

^d^ the percentage of twin deliveries where twin A is born vaginally (vag) and twin B via urgent CS

The incidence of perineal I, II and III degree tears was 0.7, 1.7 and 0.4%, respectively, during the study period with rather similar numbers for singletons (0.2, 0.7 and 0.3%). Perineal I and II degree tears showed an increasing trend, but the incidence of III degree tears remained stable. No IV degree tears were reported.

Postpartum haemorrhage (including clotting disorders, third stage bleeding, late or other bleeding related to labour) in twin mothers has increased significantly from 3.3 to 12.6% (*p* = 0.001) during the study period and is higher than among singletons (1.3%) (Fig. 3). Placenta was praevia in 1.5% of twin pregnancies (range 0.4–3.3%). Abruption of placenta occurred in 0.9% of twin pregnancies (range 0.4–2.1%). Thromboembolic events, including deep venous thrombosis, pulmonary/cerebrovascular embolism, and other/unspecified thrombosis were reported in 0.4% of twin pregnancies.

No maternal deaths were reported among twin mothers. In general, maternal deaths are very rare in Finland (1–4 per year). Perinatal mortality decreased markedly from 41.9 to 6.5 per 1000 for twin A and from 52.7 to 15.6 per 1000 for twin B (*p* < 0.0001).

## Discussion

Our purpose was to create an outline of twin pregnancies in Finland, with emphasis on maternal complications. From 1994 to 2000, the proportion of twin births increased from 1.1 to 1.7%, simultaneously with the enhanced use of ART, but also due to postponed childbearing [7]. Similar trends have been reported by Ananth and Chauhan [14]. After shifting towards a general policy to avoid multiple pregnancies in ART, twins have accounted for 1.3–1.4% of all births.

In our study, twin parturients with advanced age were a growing group. Higher age correlates with increased CS rates, also in our material, and predisposes to pregnancy complications and thus, preterm delivery [6, 7]. The combination of advanced age and existing risk factor(s) (e.g. smoking or obesity) accumulate the risks for several complications [15].

In Finland, uncomplicated dichorionic twins are usually delivered at 38–40 weeks, monochorionic at 36–38 weeks, not only depending on hospital guidelines but also on expert opinions. Differences also exist compared to international guidelines, necessitating further evaluation of the protocols in Finland [5]. In this study, preterm deliveries increased from 40.5 to 49.3%, likely due to iatrogenic prematurity. This is, however, lower than has been previously reported [14].

The increased placental mass predisposes to overproduction of angiostatic factors and thus to placental hypoxia [16]. Obesity, diabetes, pre-existing vascular diseases, and oocyte donation add to the several risk factors for developing pre-eclampsia, which generally has a worse prognosis among twin parturients [1, 2, 11]. In our study, the proportion of pre-eclamptic twin mothers rose from 4.3 to 18.1% during 1996–2006. Declining trends among singletons from Europe and Australia have been reported, but no similar data exist on twins [17]. In our study the lower PIH might, however, reflect a shift of diagnoses from plain hypertension to pre-eclampsia or more accurate use of the ICD-10 codes.

Globally, the prevalence of GDM is rising [18]. In 2008, the Finnish guidelines on oral glucose tolerance test were tightened increasing GDM diagnoses. This rise was particularly seen in twin pregnancies, probably due to meticulous testing in a high-risk pregnancy. Furthermore, advanced maternal age and obesity among twin mothers may play a role.

Due to hormonal overload to the liver, intrahepatic cholestasis of pregnancy is more common with twins; 5.5% compared to 0.4% in singletons in our material. High levels of bile acids carry a risk for intrauterine death, associating also with spontaneous preterm delivery. Follow-up, medication and induction of labour are often required, like with pre-eclampsia and GDM [19].

More complications are to be expected in twin deliveries. Both vacuum extractions and particularly urgent CSs increased steadily during our study period. Underlying reasons may be simultaneous increase in inductions, misestimated requisites for vaginal delivery or entering labour before the scheduled CS. In previous reports, the proportion of pre-labour CS is up to 56% in very high resource settings, making Finland’s elective CS rates exceptional [20]. Current or previous CS is possibly a risk factor for PPH and treatment of massive bleeding further increases maternal morbidity and mortality. Similarly with reports of rising global trends, PPH showed a steady increase in our study [21, 22]. Difficulties in estimating blood loss and misclassification of PPH diagnosis are, however, known problems in reporting excessive bleeding. Despite the low maternal mortality in developed countries, morbidity and mortality due to treatable conditions have not decreased [12, 23].

Fetal distress can develop suddenly during childbirth and predispose to operative delivery. Particularly in case of prolonged interval between births, imminent asphyxia or malpresentation may sometimes result in combined vaginal-CS [24]. In our study, approximately 1.8% of twin births were combined vaginal-CS. Previously, rates of 4.3 to 17% have been reported [24, 25]. Coming last, twin B is more prone to complications, resulting in the persistent difference in perinatal mortality between twins [26, 27]. Perinatal and neonatal outcomes are reported in detail in our upcoming research.

The strength of our study is the extensive material of a 28-year period. Previous reports supporting our notion that increased twinning follows the enhanced use of ART, exist, but less has been reported regarding pregnancy complications and maternal profiles [12]. The global increase in several pregnancy complications and postponed childbearing in developed countries are seen among twin parturients also in Finland [15, 18, 21]. As limitations, we were unable to separate chorionicity as it was not recorded in the registers. Inaccurate filling of registers may cause bias, although data compiling in Finland is considered reliable. We aim to produce national guidelines to further improve the management and outcome of twin pregnancies and help in the guidance of future parents considering ART.

## Conclusions

The results of our extensive register-based material support the notion that carrying twins predisposes to several pregnancy complications, some of which show rising trends. Some risks are related to the physiology of a twin uterus and placentation, but advanced maternal age, increasing obesity and induction of labour further predispose to complications during pregnancy and childbirth. Twin pregnancies are always high-risk pregnancies and yet more research is needed to improve their overall outcome, possibly with the help of national and international guidelines on monitoring and delivery of twins.

## Data Availability

The complete set of tables used during the current study are available from the corresponding author on reasonable request.

## Abbreviations

ART: Assisted reproductive technology
CS: Cesarean section
GDM: Gestational diabetes
ICD: International statistical classification of diseases and related health problems
PIH: Pregnancy-induced hypertension
PPH: Post partum haemorrhage
SD: Standard deviation
