# Correction to: Maternal complications in twin pregnancies in Finland during 1987–2014: a retrospective study

**DOI:** 10.1186/s12884-020-03119-z

**Published:** 2020-07-29

**Authors:** Annu-Riikka S. Rissanen, Riina M. Jernman, Mika Gissler, Irmeli Nupponen, Mika E. Nuutila

**Affiliations:** 1 Obstetrics and Gynecology, University of Helsinki and Welfare District of Päijät-Häme, Keskussairaalankatu 7, 15850 Lahti, Finland; 2 Obstetrics and Gynecology, University of Helsinki and Helsinki University Hospital, Haartmaninkatu 2, P.O. BOX 140, 00029 Helsinki, HUS Finland; 3 Finnish Institute for Health and Welfare, P.O. BOX 30, 00271 Helsinki, Finland; 4 Department of Neurobiology, Care Sciences and Society, Karolinska Institute, Stockholm, Sweden; 5 Children’s Hospital, University of Helsinki and Helsinki University Hospital, Stenbäckinkatu 11, P.O. BOX 281, 00029 Helsinki, HUS Finland

**Correction to: BMC Pregnancy Childbirth 19, 337 (2019)**

**https://doi.org/10.1186/s12884-019-2498-x**

Following publication of the original article [1], the authors identified some errors in Methods, Results and Discussion sections.

The Methods, Results and Discussion sections have been replaced with the updated version and the changes have been highlighted in **bold typeface**.

## Methods

### Statistical analyses

The data were analysed using SPSS (IBM SPSS Statistics for Windows, Version 24.0, Armonk, NY, IBM Corporation) **and RStudio (RStudio: Integrated Development Environment for R 1.1.447, Inc., Boston, MA 2016)**. Assessment of the normal distribution of variables was done with the Shapiro-Wilk test and a *p*-value < 0.05 with a 95% confidence interval was considered statistically significant. To compare medians of the variables, related samples Wilcoxon signed rank test was used. One sample t-test was used to compare means of variables. **Chi-squared test to compare groups and two sample proportions z-test to compare proportions were also performed**. Microsoft Excel 2010 and SPSS 24.0 were used to create figures, graphs and trend lines. The results are expressed in percentages, and the means, medians, ranges and standard deviations (SD) are reported when appropriate.

## Results

Paragraph 5, sentence 1.

The incidence of pre-eclampsia (range 4.3–18.1%) increased significantly (***p*** **< 0.001**) from 1996 to 2006, after which it has been 13.3–17.6% (Fig. 3).

Paragraph 6, sentence 1.

Gestational diabetes increased from 3.3 to 20.7% (***p*** **<** 0.001, average 10.4%) among twins.

Time and mode of delivery and complications.

Paragraph 4, sentences 1, 2 and 3.

Twin A was born via CS in 45.3% and twin B in 47.1%. A little less than half of CSs were elective (mean 21.4% until 2001, after which a decrease from 23.6 to 17.8% was seen (***p*** **= 0.004**). Opposite to the share of elective CS, the proportion of all CS has risen during the reported years 1991–2014 (*p* = **0.006**).

Paragraph 7, sentence 1.

Postpartum haemorrhage (including clotting disorders, third stage bleeding, late or other bleeding related to labour) in twin mothers has increased significantly from 3.3 to 12.6% (***p*** **<** 0.001) during the study period and is higher than among singletons (1.3%) (Fig. 3).

## Discussion

Paragraph 1, sentence 2.

From **1987 to 1998**, the proportion of twin births increased from 1.1 to 1.7%, simultaneously with the enhanced use of ART, but also due to postponed childbearing [7].
